# Impact of Preoperative Conjunctival Vascular Area on Surgical Outcomes in Trabeculectomy with Mitomycin C for Glaucoma: A Comprehensive Analysis

**DOI:** 10.3390/vision9030058

**Published:** 2025-07-14

**Authors:** Yasunari Hayakawa, Takayuki Inada

**Affiliations:** 1 Urawa Central Eye Institution, 2492 Ooyaguchi, Saitama 336-0042, Japan; 2 Kawagoe Central Eye Institution, Kawagoe 350-1122, Japan; 3 Koshigaya Central Eye Institution, Koshigaya 343-0041, Japan; 4 Kumagaya Central Eye Institution, Kumagaya 360-0833, Japan; 5 Yorii Central Eye Institution, Yorii 369-1202, Japan; 6 Onohara Eye Institution, Chichibu 368-0005, Japan; 7 Kisarazu Central Eye Institution, Kisarazu 292-0823, Japan

**Keywords:** glaucoma, trabeculectomy, bleb

## Abstract

Trabeculectomy with mitomycin C is a key surgical intervention for managing glaucoma when conservative treatments fail. The success of trabeculectomy is influenced by various factors, including preoperative ocular characteristics like conjunctival vascularity. This study aims to explore the relationship between the preoperative conjunctival vascular area and post-trabeculectomy outcomes in glaucoma patients. By analyzing the conjunctival vascular density, intraocular pressure (IOP), bleb morphology, laser suture lysis (LSL) frequency, and postoperative eye drops, this research sheds light on the impact of preoperative vascularity on surgical success. Results show that lower preoperative conjunctival vessel density is associated with favorable outcomes, such as better bleb formation and reduced need for postoperative interventions, while higher conjunctival vessel density correlates with complications like hyphema. These findings emphasize the importance of assessing preoperative conjunctival vascularity to optimize trabeculectomy outcomes and personalize treatment strategies for glaucoma patients.

## 1. Introduction

Glaucoma is a leading cause of irreversible blindness globally, characterized by progressive optic nerve damage and visual field loss [1,2,3]. It poses a significant public health challenge, impacting individuals worldwide with its insidious progression and debilitating consequences on visual function. The management of glaucoma entails a multifaceted approach involving various treatment modalities, ranging from topical medications to laser procedures and surgical interventions [4,5,6]. Among these treatment options, trabeculectomy with mitomycin C stands out as a cornerstone surgical procedure utilized in cases where conservative measures fail to adequately control intraocular pressure [7,8,9]. By creating a new filtration pathway for aqueous humor drainage, trabeculectomy aims to alleviate the elevated intraocular pressure burden that contributes to disease progression in glaucomatous eyes [10,11]. Despite its proven efficacy in lowering intraocular pressure and preserving visual function, trabeculectomy outcomes can exhibit variability influenced by a multitude of preoperative, intraoperative, and postoperative factors.

The success of trabeculectomy is contingent not only on the surgical technique and postoperative management but also on the preoperative ocular characteristics that set the stage for the surgical procedure. The conjunctiva, a thin and transparent membrane covering the ocular surface, plays a pivotal role in trabeculectomy outcomes by influencing the formation and function of the filtration bleb, a critical component in maintaining long-term intraocular pressure control postoperatively [12,13]. Among the various preoperative factors that can impact trabeculectomy success, the vascularity of the conjunctiva emerges as a key determinant deserving closer examination. Conjunctival vascular characteristics, including the density, tortuosity, and caliber of vessels within the conjunctiva, are believed to have implications for bleb morphology, wound healing dynamics, and postsurgical outcomes following trabeculectomy with mitomycin C [14,15]. Therefore, understanding the influence of preoperative conjunctival vascular features on surgical results is essential in optimizing treatment strategies and enhancing visual outcomes for glaucoma patients undergoing trabeculectomy procedures.

In light of the intricate interplay between preoperative conjunctival vascularity and trabeculectomy outcomes, this study endeavors to delve deeper into the relationship between the preoperative conjunctival vascular area and postoperative clinical parameters in patients undergoing trabeculectomy with mitomycin C for glaucoma. By comprehensively analyzing preoperative conjunctival vascular characteristics and their impact on surgical success, this research aims to provide valuable insights into personalized treatment approaches tailored to individual conjunctival profiles and optimize therapeutic outcomes in the management of glaucoma.

## 2. Material and Methods

### 2.1. Ethical Approval

This retrospective cohort study was approved by our institution’s committee (Tohankai Eye Institution’s Ethics Committee, approval number 0002, 31 January 2023) and adhered to the regulations of clinical practice and the tenets of the Declaration of Helsinki. The authors had access to information that could identify individual participants during and after data collection. Informed consent has been obtained from all participants in th study. The data for our research purposes were accessed from 1 February 2024. During the data collection process, the authors did not have access to any information that could identify individual participants. All data were anonymized to maintain confidentiality.

### 2.2. Study Design

This was a retrospective cohort study. Glaucoma patients underwent trabeculectomy with 0.04% mitomycin C (MMC) by Hayakawa Y. at Urawa Central Eye Institution, Chiba Central Eye Institution, Koshigaya Central Eye Institution, Kumagaya Central Eye Institution, Yorii Central Eye Institution, Onohara Eye Institution, and Kisarazu Central Eye Institution, between 1 January 2020, and 31 December 2023.

### 2.3. Inclusion and Exclusion Criteria

This study enrolled participants aged 18–90 years who were diagnosed with mild-to-severe glaucoma by a glaucoma specialist. The diagnosis was based on specific criteria for the following types of glaucoma:

1. Primary Open-Angle Glaucoma (POAG):
  - Characteristic glaucomatous optic neuropathy.
  - Confirmation through two reliable visual field tests demonstrating repeatable glaucomatous defects.
  - Open-angle observed on gonioscopy.
  - Intraocular pressure (IOP) exceeding 21 mmHg on two consecutive visits using a Goldmann applanation tonometry.
2. Pseudoexfoliation Glaucoma (PEG):
  - Diagnosis was made based on the observation of white dandruff-like material deposited in the trabecular meshwork, as confirmed by gonioscopy.
3. Combined Mechanism Glaucoma (CMG):
  - Patients who do not meet the criteria for typical Primary Open-Angle Glaucoma (POAG) or chronic angle-closure glaucoma (CACG) are categorized as CMG. This classification includes individuals exhibiting characteristics of both open and closed angles. Specifically, anterior chamber angle findings may show partial closure or plateau configuration, leading to a unique presentation of glaucoma that does not strictly align with the definitions of either POAG or CACG.

The exclusion criteria were secondary glaucoma and neovascular glaucoma.

### 2.4. Surgical Procedure

For fornix-based trabeculectomy, a limbal peritomy centered at the upper temporal position (superotemporal quadrant) was created. The conjunctiva and Tenon’s capsule were dissected using Westcott scissors, and a 0.04% MMC-soaked cellulose sponge was applied for 3 min, according to the surgeon’s preference. A partial-thickness scleral flap was created, followed by sclerotomy and iridectomy in the same fashion as limbus-based trabeculectomy. The scleral flap was closed with a relatively loose closure using 4 interrupted 10-0 nylon sutures. The aim was to allow controlled aqueous outflow. At the conclusion of the surgery, bleb formation was confirmed by injecting balanced salt solution (BSS) into the anterior chamber. The conjunctiva and Tenon’s capsule were closed using two 10-0 nylon sutures at the limbus. As stated in the study design, all procedures were performed by a single surgeon, Hayakawa Y., ensuring consistency in surgical technique. All participating facilities belong to the same medical corporation, which ensures consistency in the surgical materials and suture types used.

### 2.5. Measurement

A retrospective cohort of 72 eyes from 72 consecutive patients who underwent primary trabeculectomy with mitomycin C for glaucoma within a specified timeframe was meticulously evaluated in this study. The patient cohort represented a diverse population with varying demographics, underlying glaucoma subtypes, and preoperative ocular characteristics, all of which were documented and analyzed for their potential influence on surgical outcomes.

**Figure 1:**
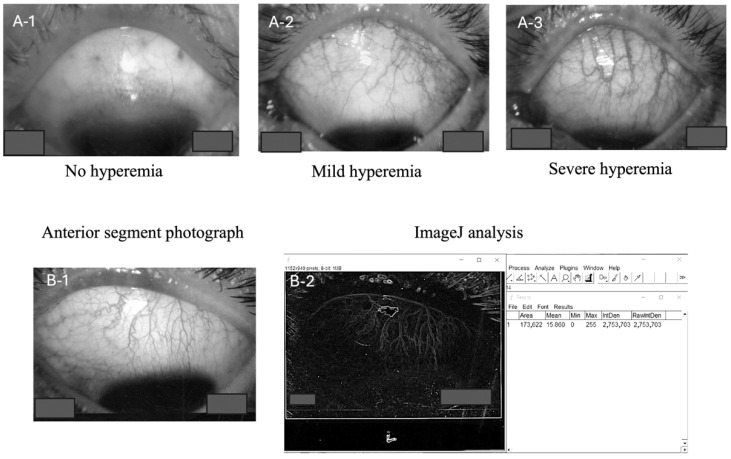
Photographs showing varying degrees of preoperative conjunctival hyperemia (none, (**A-1**); mild, (**A-2**); severe, (**A-3**)). Anterior segment images were captured, converted to TIFF files (**B-1**), and imported into ImageJ. The images were then converted to 8-bit. Following this, outlines were traced, and a region of interest was defined to match the conjunctiva. The Integrated Density value was used as the measurement parameter (**B-2**).

**Figure 2:**
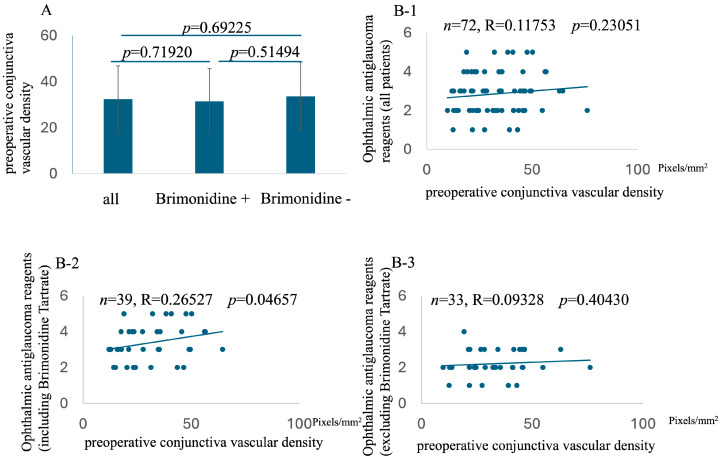
Comparison of preoperative conjunctival vessel density among all patients, patients on brimonidine tartrate, and patients not on brimonidine tartrate (**A**). Relationship with preoperative conjunctiva vascular density and preoperative number of ophthalmic antiglaucoma reagents (including all reagents, (**B-1**); including brimonidine tartrate, (**B-2**); excluding brimonidine tartrate, (**B-3**)).

**Figure 3:**
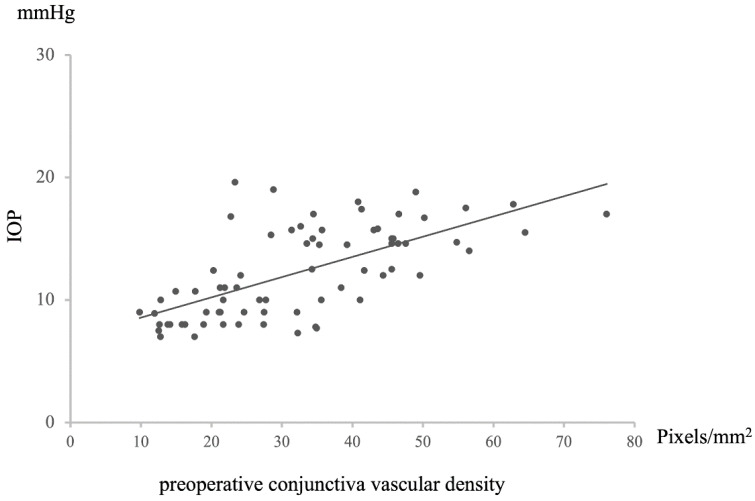
Relationship between preoperative conjunctival vascular density and IOP at 12 M after surgery. Statistical analysis was performed using the Pearson’s correlation coefficient. *n* = 72, R = 0.65158, *p* = 5.70321 × 10^−10^.

Representative preoperative conjunctival images demonstrating different levels of hyperemia are shown in Figure 1A-1–A-3. This figure includes examples of eyes exhibiting no hyperemia, mild hyperemia, and pronounced hyperemia. Each measurement was conducted under consistent conditions with a single observer performing all analyses to ensure the uniformity and reproducibility of the findings.

Prior to trabeculectomy (measurements performed within one week preoperatively), the conjunctival vascular density was measured using ImageJ software (version 1.0.0) according to the following standardized protocol: Photographs of the anterior segment were captured using the same slit lamp microscope (Haag-Streit AG, Baden, Switzerland) at a magnification of 6.3×. Images were captured using diffuse illumination in a darkened room. A slit beam was not used; rather, diffuse light was employed to illuminate the conjunctival vessels evenly. Images were focused specifically on the superior temporal quadrant where the trabeculectomy was performed. All images were saved as TIFF files and subsequently transformed into 8-bit black and white images using ImageJ software (NIH, Madison, WI, USA) for accurate measurement (Figure 1B-1). Patients were not instructed to discontinue the use of topical medications, including prostaglandin analogs and brimonidine tartrate, prior to imaging or surgery.

In addition to these measurements, the number and type of ophthalmic antiglaucoma reagents used preoperatively were recorded for each patient, with the number of reagents quantified as an ophthalmic antiglaucoma reagent score. This included a specific assessment of brimonidine tartrate use, given the potential association between this medication and conjunctival vascular density.

Postoperatively, patients were followed up for a minimum of 12 months to monitor their clinical progress and assess various parameters indicative of surgical success or potential complications. The key postoperative parameters evaluated in this study included intraocular pressure measurements at the 12-month mark, the rate of intraocular pressure reduction from baseline, the morphological characteristics of filtration blebs, the frequency of laser suture lysis (LSL) procedures, alterations in glaucoma medication regimens post-trabeculectomy, the occurrence and resolution of hyphema, and the occurrence of flat anterior chamber (FAC).

IOP was measured using Goldman applanation tonometer (GAT). The ratio of IOP reduction, calculated as (preoperative IOP–postoperative IOP/preoperative IOP) × 100, was also a critical parameter assessed in this study.

In classifying the post-trabeculectomy blebs, a simplified classification based on Migdal and Hitchings’ classification [16] was used and customized as follows: Grade 1, thin and transconjunctival flow of aqueous humor resulting in good filtration (avascular sub-Tenon’s bleb); Grade 2, thin, diffuse, relatively avascular bleb with microcysts resulting in good filtration; Grade 3, flat, non-microcystic bleb with engorged vessels resulting in non-filtration bleb; and Grade 4, localized, highly elevated, engorged vessels, cyst-like or hypertrophied Tenon’s resulting in encapsulated bleb. The blebs were classified under slit lamp examination at the subconjunctival lump at 12 months postoperatively. The MBGS (Moorfields Bleb Grading System) and IBAGS (Indiana Bleb Appearance Grading Scale) are commonly used for classifying blebs [17,18,19]. However, in this study, we chose not to use these classifications, as we felt that they were too detailed for the scope of our study, making it difficult to detect significant differences within the sample size.

The presence or absence of a FAC was confirmed by slit lamp examination, and FAC grading of 1–3 was classified as FAC+ [20,21].

For cases receiving preoperative antithrombotic agents, administration was halted. In this study, all cases of hyphema were graded as less than Grade 1, therefore classified as − for absence of hyphema and + for presence of hyphema.

### 2.6. Statistical Analysis

To analyze the relationship between the preoperative conjunctival vascular area and several postoperative outcomes, we employed a range of statistical methodologies. Logistic regression models were utilized to assess the impact of the preoperative conjunctival vascular area on the occurrence of FAC and hyphema. For these models, the preoperative conjunctival vascular area was the independent variable, and the presence or absence of FAC or hyphema was the dependent variable. These were univariate logistic regression models. Additionally, the Pearson’s correlation coefficient was calculated to explore the linear relationship between the preoperative vascular area and the rate of intraocular pressure reduction. Spearman’s rank correlation coefficient was applied to assess the relationships among categorical variables such as bleb morphology classification and postoperative number of eye drops administered.

Analyses were conducted using Stat Plus (AnalystSoft Inc., Alexandria, VA, USA) to ensure the robustness methods and adequately addresses the complexities of data.

## 3. Results

### 3.1. Demographic

Seventy-two eyes with POAG, PEG, and CMG underwent trabeculectomies. A summary of the patient data and a compilation of the measured values are presented in Table 1. Data analysis revealed a series of noteworthy associations between preoperative conjunctival vascular density and various postoperative parameters, shedding light on the intricate relationship between conjunctival vascularity and surgical outcomes in patients undergoing trabeculectomy with mitomycin C for glaucoma.

### 3.2. Relationship Between Conjunctival Vascular Density, IOP, Bleb Morphology, LSL, and Pre- or Postoperative Glaucoma Eye Drops

While no significant correlation was found between preoperative conjunctival vascular density and the overall ophthalmic antiglaucoma reagent score or the score excluding brimonidine tartrate, a significant positive correlation was observed between preoperative conjunctival vascular density and the ophthalmic antiglaucoma reagent score, specifically in patients using brimonidine tartrate (*n* = 39, R = 0.26527, *p* = 0.04657, Figure 2). This indicates that in patients using brimonidine tartrate preoperatively, higher conjunctival vascular density was associated with a greater number of ophthalmic antiglaucoma reagents needed postoperatively.

**Figure 4:**
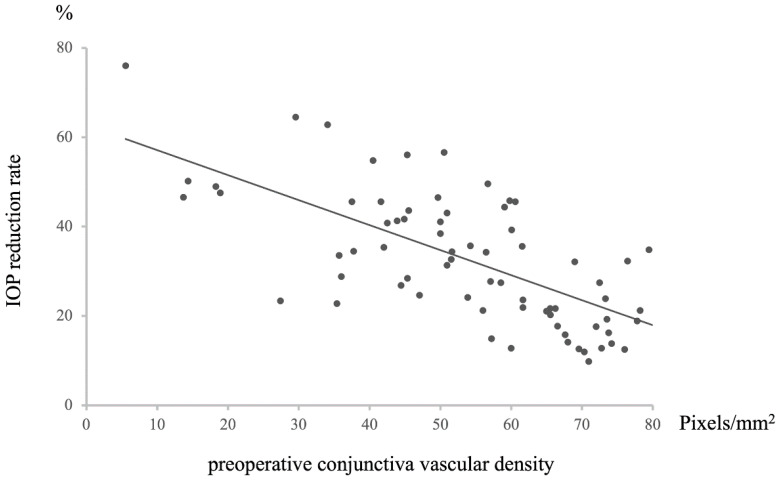
Relationship between preoperative conjunctival vascular density and IOP reduction rate at 12 M after surgery. Statistical analysis was performed using the Pearson’s correlation coefficient. *n* = 72, R = −0.6699072, *p* = 1.23476 × 10^−10^.

A positive correlation was observed between preoperative conjunctival vessel density and postoperative intraocular pressure (IOP) (*n* = 72, Rho = −0.67446, *p* = 8.29203 × 10^−11^, Figure 3). Specifically, a higher preoperative conjunctival vessel density was associated with higher IOP values at 12 months postoperative, while a lower preoperative conjunctival vessel density was associated with lower IOP values. This relationship extended to a negative correlation between preoperative conjunctival vessel density and the rate of IOP decrease at 12 months postoperative (*n* = 72, R = −0.6699072, *p* = 1.23476 × 10^−10^, Figure 4). In essence, a higher preoperative conjunctival vessel density was linked to a lower rate of IOP decrease over 12 months, while a lower preoperative conjunctival vessel density was associated with a higher rate of IOP decrease.

**Figure 5:**
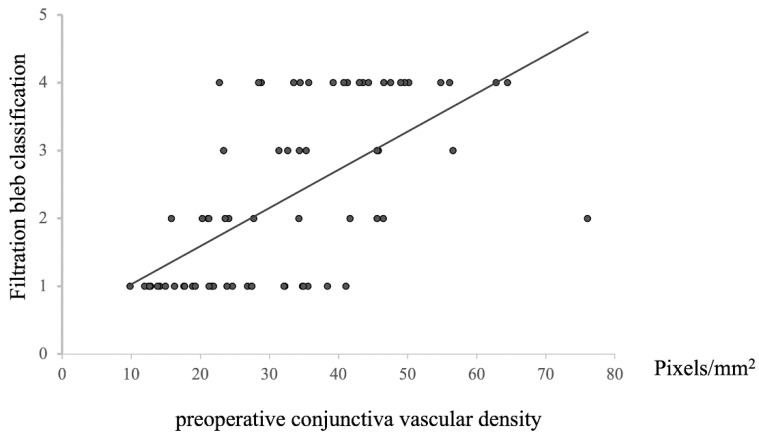
Relationship between preoperative conjunctival vascular density and filtration bleb classification at 12 M after surgery. Statistical analysis was performed using the Spearman’s correlation coefficient. *n* = 72, Rho = −0.67446, *p* = 8.29203 × 10^−11^.

A positive correlation was observed between the preoperative conjunctival vessel density and bleb morphology at 12 months postoperative (*n* = 72, Rho = −0.67446, *p* = 8.29203 × 10^−11^, Figure 5). This finding indicated that higher preoperative conjunctival vessel density led to higher rankings in bleb classification, whereas lower preoperative vessel density resulted in lower rankings. Given that lower rankings imply better filtration of bleb morphology, it was found that lower preoperative conjunctival vessel density could facilitate the formation of more favorable blebs.

**Figure 6:**
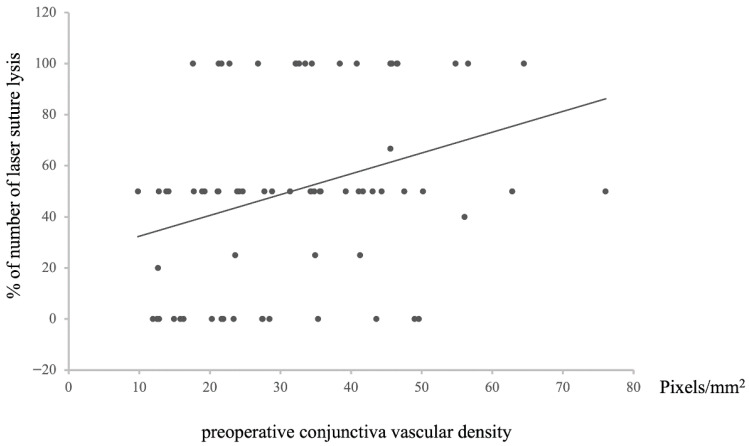
Relationship between preoperative conjunctival vascular density and the percentage of laser suture lysis after surgery. Statistical analysis was performed using the Spearman’s correlation coefficient. *n* = 72, Rho = −0.31976, *p* = 0.00618.

Furthermore, a positive correlation was noted between preoperative conjunctival vessel density and the frequency of undergoing LSL (*n* = 72, Rho = −0.31976, *p* = 0.00004, Figure 6). This result aligns with previous findings related to postoperative IOP and bleb classification, maintaining a consistent pattern across relationships.

**Figure 7:**
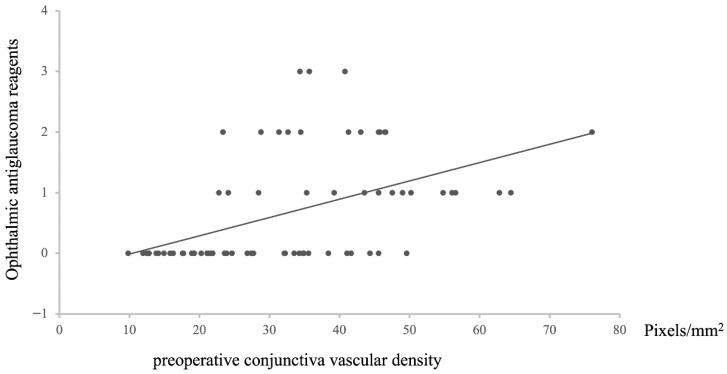
The relationship between preoperative conjunctival vascular density and number of ophthalmic antiglaucoma reagents after surgery. Statistical analysis was performed using the Spearman correlation coefficient. *n* = 72, Rho = −0.57631, *p* = 1.17487 × 10^−7^.

Regarding the relationship between the preoperative conjunctival vascular area and the postoperative intraocular pressure, the preoperative conjunctival vascular area was positively correlated with the number of postoperative ophthalmic antiglaucoma reagents (*n* = 72, Rho = −0.57631, *p* = 1.17487 × 10^−7^, Figure 7).

### 3.3. The Relationship Between Conjunctival Vascular Density, Hyphema, and FAC

In addition, logistic regression analysis revealed an association between preoperative conjunctival vessel density and postoperative occurrence of hyphema (*n* = 72, *p* = 0.00299, Table 2). However, no significant association was found between the preoperative conjunctival vessel density and FAT (*n* = 72, *p* = 0.08261, Table 2).

Relationship between preoperative conjunctival vascular density and postoperative flat anterior chamber thickness Statistical analysis was performed with logistic regression models. *n* = 72, *p* = 0.08261.

## 4. Discussion

This study investigated the relationship between preoperative conjunctival vessel density and postoperative outcomes following trabeculectomy. Our findings indicate that a lower preoperative conjunctival vessel density, suggestive of reduced conjunctival congestion, is generally associated with improved surgical outcomes. Specifically, lower density correlated with superior bleb morphology, decreased incidence of late surgical failure (LSF), reduced intraocular pressure (IOP), and decreased reliance on IOP-lowering medications. These results are consistent with prior research demonstrating the influence of conjunctival vascularity on postoperative healing and bleb morphology [22]. Furthermore, and importantly, our study revealed a significant association between preoperative conjunctival vascular density and postoperative ophthalmic antiglaucoma reagent usage, particularly among patients using brimonidine tartrate. In this subgroup, higher preoperative conjunctival vascular density was significantly associated with a greater number of postoperative glaucoma medications. In particular, concerning common postoperative complications, we observed a positive association between higher preoperative conjunctival vessel density and the occurrence of hyphema.

Previous studies utilizing AS-OCT angiography have examined the relationship between postoperative conjunctival vessel density, bleb formation, and IOP following trabeculectomy and PreserFlo microshunt implantation [23,24,25]. However, this study provides a novel contribution by directly assessing the correlation between preoperative conjunctival vessel density and a range of postoperative parameters. Investigations focused on this preoperative relationship remain limited, making our findings a valuable addition to the existing literature. While this study did not directly assess conjunctival vessel density prospectively, reports have indicated that preoperative deepening of the upper eyelid sulcus (DEUS) or Prostaglandin-Associated Periorbitopathy (PAP) is associated with postoperative outcomes following trabeculectomy [26]. These observations, which often correlate with greater preoperative IOP-lowering medication use, are consistent with our findings, suggesting that increased conjunctival congestion, potentially driven by prostaglandin analogs, is associated with less favorable surgical outcomes [27].

The significant finding regarding brimonidine tartrate suggests a possible link between its use and increased conjunctival vascularity, potentially contributing to poorer surgical outcomes. While the exact mechanism remains unclear, brimonidine tartrate is known to have vasoconstrictive and inflammatory effects, which might paradoxically lead to increased conjunctival vascular congestion in the long term [28,29]. Therefore, our results emphasize the importance of carefully evaluating the necessity of each preoperative glaucoma medication and considering the potential impact of brimonidine tartrate on conjunctival vascularity and subsequent trabeculectomy success.

A key limitation of this study was the absence of AS-OCT angiography for conjunctival vessel density measurements. While AS-OCT angiography represents a more sophisticated approach, it is also a resource-intensive technology that is not universally available [30,31]. Our methodology, involving image capture, conversion, and quantification using ImageJ software, offers a more accessible and readily implementable alternative for approximating conjunctival vascularity in diverse clinical settings. However, it is crucial to acknowledge that ImageJ is inherently semi-quantitative and subject to observer bias. Despite its inherent limitations compared to AS-OCT angiography, this simplified approach provides a feasible means of roughly quantifying conjunctival vascularity, potentially informing clinical decision-making. Future research should prospectively incorporate measures of intra-observer consistency to rigorously assess the reliability of this ImageJ-based methodology. The results should be interpreted with caution given the potential for variability and subjectivity.

Another limitation of our study is the subjective classification of bleb morphology at 12 months. We acknowledge that AS-OCT provides a more detailed and quantitative assessment of bleb structure and correlates strongly with surgical success, as demonstrated in prior studies [32]. The lack of AS-OCT is a limitation. However, AS-OCT (particularly swept-source OCT) was not uniformly available across all sites, and the logistical challenges of implementing AS-OCT at all locations would have significantly impacted the feasibility of conducting this retrospective study. Furthermore, we aimed to maintain consistency in our evaluation methods throughout this study. As the preoperative conjunctival vascular density was assessed through photographic analysis, we opted to evaluate postoperative bleb morphology using a similar photographic approach. This ensured that both preoperative and postoperative parameters were evaluated using a consistent methodology, reducing potential bias introduced by different assessment techniques. Future studies should incorporate AS-OCT to provide a more comprehensive evaluation of bleb morphology and its relationship to surgical outcomes.

Furthermore, we acknowledge the potential influence of confounding factors, including patient age, glaucoma duration, and the use of IOP-lowering medications (aside from the identified influence of brimonidine tartrate), on postoperative outcomes. The established impact of these variables on both clinical presentation and treatment response necessitates their careful consideration when interpreting our results. We also recognize the potential value of correlating not just preoperative but also postoperative conjunctival vessel density with clinical outcomes. While assessing postoperative conjunctival vessel density could provide additional insights, our current study focuses primarily on preoperative and immediate postoperative outcomes to establish a foundational understanding. Although we did not assess conjunctival vessel density postoperatively because we consider the morphological evaluation of the bleb to be partially related to vessel density, future research should aim to incorporate serial postoperative assessments of conjunctival vessel density, possibly using AS-OCT angiography, to build upon these findings. Future research should aim to control for these confounding variables through rigorous study design and statistical analysis in order to further elucidate the independent relationship between preoperative conjunctival vascular density and glaucoma surgery outcomes. Integrating more comprehensive assessments and controlling for these factors will strengthen our understanding of the underlying mechanisms and enhance the robustness of future findings. Furthermore, we recognize that the duration of preoperative glaucoma medication use may also influence postoperative outcomes, potentially through its effect on conjunctival vascularity. While our data collection limitations precluded a robust analysis of this factor, future studies should investigate the relationship between the duration of preoperative topical medication use and trabeculectomy success.

It is also important to acknowledge the potential confounding effect of glaucoma diagnosis (POAG, PEG, CMG) on our findings. While our cohort included a mix of glaucoma subtypes, the number of cases of CMG and PEG was limited compared to POAG. This difference in sample sizes may have limited our ability to perform stratified analysis by diagnosis. While we acknowledge that concentrating on POAG might reduce biological noise, our rationale for including CMG and PEG was their unique contribution to the spectrum of conjunctival vascular changes in glaucoma. CMG and PEG patients in our cohort often presented with advanced disease and a history of extensive topical medication use, leading to pronounced conjunctival hyperemia, which was a key factor in our vascular density analysis. Furthermore, we believe that excluding these patients would reduce the clinical relevance of our findings, as advanced glaucoma cases with complex medication histories are frequently encountered in real-world practice. The different disease states could affect the conjunctival vascularity that may affect postoperative outcomes. Future studies with larger cohorts and a more balanced representation of glaucoma subtypes are needed to fully address this issue.

Regarding the statistical analysis, we acknowledge the reviewer’s suggestion to include confidence intervals and apply multiple testing corrections. Given the exploratory nature of this study and our aim to identify potential associations, we opted to retain our original statistical approach. We recognize that this decision carries the risk of Type I errors, and therefore, the *p*-values presented should be interpreted cautiously. The findings should be considered preliminary until validated in independent cohorts. Furthermore, while we employed logistic regression models to assess the impact of the preoperative conjunctival vascular area on FAC and hyphema, these were univariate models, which limits our ability to account for other potential confounding variables. Future studies should incorporate multivariate models to provide a more comprehensive assessment. Acknowledging these statistical considerations, we reiterate that our findings warrant further investigation and confirmation in larger, more controlled studies.

Furthermore, the inclusion of postoperative IOP-lowering medications introduces a potential bias in the assessment of true IOP reduction. While washed-out IOP readings would provide a more isolated measure of surgical efficacy, we chose to present the IOP reduction rates with medication, as this represents the overall clinical outcome and the real-world effect of the surgery, including the effect of any necessary adjunctive medical therapy. We recognize the limitation this poses to the interpretation of surgical efficacy alone, and we will add a discussion of this point, clarifying that our results reflect the combined effect of surgery and adjunctive medical therapy.

Moreover, although we did not observe any cases of bleb leak in patients who developed FAC, over-filtration was the most likely contributing factor in our cases. Our analysis did not demonstrate a statistically significant association between preoperative conjunctival vascular density and the occurrence of FAC, but it is possible that a trend exists that we were unable to detect with our sample size. It is also possible that other factors not directly assessed in this study, such as scleral flap tightness and internal ostium size, had a greater influence on the development of FAC.

In conclusion, our findings suggest that lower preoperative conjunctival vessel density is generally associated with improved outcomes following trabeculectomy. However, our results also highlight the importance of carefully considering the influence of preoperative glaucoma medications, particularly brimonidine tartrate, on conjunctival vascularity and subsequent surgical success. Given the potential for brimonidine tartrate to increase preoperative conjunctival vessel density and negatively impact postoperative outcomes, we propose that ophthalmic antiglaucoma reagents should be appropriately managed before surgery. This may involve reviewing and adjusting medication regimens to optimize the conjunctival environment and potentially improve trabeculectomy outcomes. Specifically, considering the potentially paradoxical effects of brimonidine tartrate, future studies should investigate the impact of a preoperative ‘washout’ period, where the medication is discontinued for a defined time before surgery, on conjunctival vascularity and subsequent trabeculectomy success. This suggestion, prompted by a thoughtful reviewer, warrants further investigation to determine if such a strategy could optimize the conjunctival environment and improve surgical outcomes. Further research is warranted to investigate the precise mechanisms by which brimonidine tartrate affects conjunctival vascularity and to determine the optimal strategies for managing preoperative glaucoma medications to maximize the success of trabeculectomy.

## Figures and Tables

**Table 1:** The summary of patient data and compilation of measured values.

Category	Group	Number
sex	male	37
	female	35
age	71.00 ± 13.76 age-old	
glaucoma type	POAG	63 (87.50%)
	CMG	5 (6.94%)
	PEG	4 (5.56%)
Preope conjunctiva vascular density	32.44 ± 14.44 pixels/mm^2^	
preope IOP	27.89 ± 5.42 mmHg	
preope number of medications	2.85 ± 1.06	
IOP at 12 M after the surgery	12.26 ± 3.65 mmHg	
IOP reduction rate	54.07 ± 17.27%	
filtration bleb classification	2.29 ±1.28	
number of LSL	50.72 ± 36.70	
postope number of medications	0.67 ± 0.90	
flat anterior chamber	+	17 (23.61%)
	−	55 (76.39%)
hyphema	+	9 (12.50%)
	−	63 (87.50%)

**Table 2:** Relationship preoperative conjunctival vascular density and post operative hyphema and narrow anterior chamber.

	Hyphema	FAC (Flat Anterior Chamber)
Chi-square	29.696	3.11362
Degrees of Freedom	1	1
Beta	0.2296	0.03385
Standard Error	0.07734	0.01950
*p*-value	0.00299	0.08261
Odds Ratio	1.25811	1.03443

## Data Availability

The datasets generated and analyzed during the current study are available from the corresponding author on reasonable request.
